# Supplementary material for: Genome analysis reveals evolutionary mechanisms of adaptation in systemic dimorphic fungi
Source: Sci Rep. 2018 Mar 14;8:4473. doi: 10.1038/s41598-018-22816-6 (PMC5852033; doi:10.1038/s41598-018-22816-6)
Supplement: Supplementary file 1 — Supplementary Information [file 41598_2018_22816_MOESM1_ESM.docx]

**Genome analysis reveals evolutionary mechanisms of adaptation in systemic dimorphic fungi**

José F. Muñoz^1^, Juan G. McEwen^2,3^, Oliver K. Clay^3,4^ , Christina A. Cuomo^1^*

^1^Broad Institute of MIT and Harvard, Cambridge, MA, United States.

^2^ Cellular and Molecular Biology Unit, Corporación para Investigaciones Biológicas, Medellín, Colombia. ^3^ School of Medicine, Universidad de Antioquia, Medellín, Colombia.

^4^ School of Medicine and Health Sciences, Universidad del Rosario, Bogotá, Colombia

* [cuomo@broadinstitute.org](mailto:cuomo@broadinstitute.org)

**List of supplementary tables**

**Table S1.** List of species included in this study

**Table S2.** Protein family, gene ontology, and pathway enrichment analyses

**Table S3.** Secondary metabolite gene clusters

**Table S4.** Known virulence factors

**Table S5.** Ortholog clusters conservation analysis

**Table S6.** Conserved genes induced during *in vivo* RNA-seq data sets

**Table S7.** Mating-type locus for sexual reproduction

**List of supplementary figures**

**Figure S1.** Overall description and comparison of the annotated genome assemblies

**Figure S2.** Completeness of annotated genomes

**Figure S3.** Guanine Cytosine whole genome frequency distributions

**Figure S4.** Whole genome diversity in Ajellomycetaceae genera

**Figure S5.** Maximum likelihood tree of Polyketide synthase (PKS) family

**Figure S6.** Mating type evolution within the non-Ajellomycetaceae Onygenales

**Figure S7.** Distribution of orthologs clusters

**Figure S1.** Overall description and comparison of the annotated genome assemblies included in this study, including genome size, number of protein-coding genes, and functional annotation (protein family domain PFAM; pathways KEGG-EC). The phylogenetic tree corresponds to the maximum likelihood tree using 2,505 core genes described in Fig. 1. Blue dots represent nodes supported by 100% of bootstrap replicates. *Es*: *Emergomyces*; *Ea*: *Emmonsia*.

**Figure S2.** Conservation of core eukaryotic genes (CEGs) using BUSCO and CEGMA across the Ajellomycetaceae species sequenced, assembled and annotated in this study (bold) and other genomes included in comparative genomic analyses. *Es*: *Emergomyces*; *Ea*: *Emmonsia*.

**Figure S3.** Guanine Cytosine whole genome frequency distributions. GC histograms of overlapping windows (128 bp) of the genome assemblies of *E. parva* UAMH130, *E. crescens* UAMH4076, *H. griseus* UAMH5409 and *P. hystricis* UAMH7299, and closely relatives within the Ajellomycetaceae family. The bin size of the histograms is approximately 0.1% GC. Horizontal axes show GC % and vertical axes show relative frequencies. The phylogenetic tree highlights transitions in genome architectures within the Ajellomycetaceae genera and species. Green and blue shaded boxes correspond to GC-poor and GC-rich regions, respectively. *Es*: *Emergomyces*; *Ea*: *Emmonsia*.

**Figure S4.** Limited and ample whole genome diversity in Ajellomycetaceae genera. **(A)** Heat-map depicts the identity of whole genome alignment using Mummer/promer for each taxon in the Ajellomycetaceae. The whole genome identity (%) is color coded from low (blue) to high (red). Each taxa has a color code (left) indicating the genera. *Es*: *Emergomyces*; *Ea*: *Emmonsia*. **(B)** Genera genetic variation inferred from branch lengths of the maximum likelihood phylogenetic tree of the family Ajellomycetaceae from Fig. 1 maximum. **(C)** Mummer/promer alignments of *E. parva* UAMH139 *vs* *E. parva* UAMH130 and *E. crescens* UAMH3008 *vs* *E. crescens* UAMH4076, and average percentage of identity for each comparison.

**Figure S5.** Maximum likelihood tree of Polyketide synthase (PKS) family showing contractions in Ajellomycetaceae dimorphic pathogenic fungi. Genes from Ajellomycetaceae species are highlighted with light blue. *Polytolypa hystricis* UAMH7299 and *Helicocarpus griseus* UAMH5409 PKs are color labeled green and blue, respectively.

**
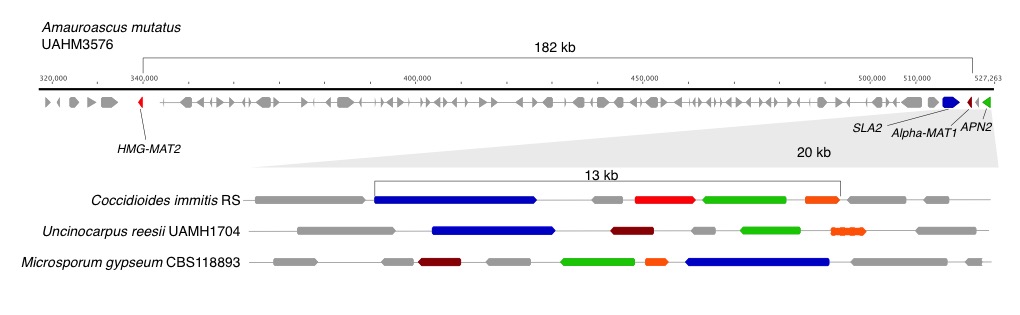
**

**Figure S6.** Mating type evolution within the non-Ajellomycetaceae Onygenales. Schema depicting orientation and conservation of the genes adjacent to the mating type locus idiomorphs HMG box (*MAT 1-2*, red) and alpha box (*MAT1-1*, dark red) in non-Ajellomycetaceae *Amaurascus mutatus*, *C. immitis*, *U. reesii*, and *M. gypseum*. In *A. mutatus*, both mating type idiomorphs are linked in the same scaffold, however they are separated by 184 kb, including 65 protein-coding genes (gray).

**Figure S7.** Distribution of ortholog clusters between Ajellomycetaceae, and other fungi included in this study. **(A)** Bar plot of orthology classes is shown, where core genes found in all genomes are shown in blue, shared genes present in more than one but not all genomes in gray, and genes that were unique to only one of the genomes in red. **(B)** Core-genome and **(C)** pan-genome gene accumulation curves for sequenced genomes from the Ajellomycetaceae.
